# Late‐Season Influenza Vaccine Effectiveness Against Medically Attended Outpatient Illness, United States, December 2022–April 2023

**DOI:** 10.1111/irv.13342

**Published:** 2024-06-23

**Authors:** Jessie R. Chung, Philip Shirk, Manjusha Gaglani, Manohar B. Mutnal, Mary Patricia Nowalk, Krissy Moehling Geffel, Stacey L. House, Tara Curley, Karen J. Wernli, Erika L. Kiniry, Emily T. Martin, Ivana A. Vaughn, Vel Murugan, Efrem S. Lim, Elie Saade, Kiran Faryar, Olivia L. Williams, Emmanuel B. Walter, Ashley M. Price, John R. Barnes, Juliana DaSilva, Rebecca Kondor, Sascha Ellington, Brendan Flannery

**Affiliations:** ^1^ Influenza Division US Centers for Disease Control and Prevention Atlanta Georgia USA; ^2^ Baylor Scott & White Health Temple Texas USA; ^3^ Department of Pediatrics Baylor College of Medicine Temple Texas USA; ^4^ Department of Family Medicine University of Pittsburgh School of Medicine Pittsburgh Pennsylvania USA; ^5^ Department of Emergency Medicine Washington University School of Medicine in St. Louis St. Louis Missouri USA; ^6^ Kaiser Permanente Washington Health Research Institute Seattle Washington USA; ^7^ Department of Health Systems Science Kaiser Permanente Bernard J. Tyson School of Medicine Pasadena California USA; ^8^ Department of Epidemiology University of Michigan School of Public Health Ann Arbor Michigan USA; ^9^ Henry Ford Health Detroit Michigan USA; ^10^ Biodesign Center for Personalized Diagnostics Arizona State University Tempe Arizona USA; ^11^ Biodesign Center for Fundamental & Applied Microbiomics Arizona State University Tempe Arizona USA; ^12^ Division of Infectious Diseases and HIV Medicine University Hospitals of Cleveland Cleveland Ohio USA; ^13^ Department of Emergency Medicine University Hospitals of Cleveland Cleveland Ohio USA; ^14^ Duke Human Vaccine Institute Duke University School of Medicine Durham North Carolina USA

**Keywords:** influenza, vaccination, vaccine effectiveness

## Abstract

**Background:**

The 2022–23 US influenza season peaked early in fall 2022.

**Methods:**

Late‐season influenza vaccine effectiveness (VE) against outpatient, laboratory‐confirmed influenza was calculated among participants of the US Influenza VE Network using a test‐negative design.

**Results:**

Of 2561 participants enrolled from December 12, 2022 to April 30, 2023, 91 laboratory‐confirmed influenza cases primarily had A(H1N1)pdm09 (6B.1A.5a.2a.1) or A(H3N2) (3C.2a1b.2a.2b). Overall, VE was 30% (95% confidence interval −9%, 54%); low late‐season activity precluded estimation for most subgroups.

**Conclusions:**

2022–23 late‐season outpatient influenza VE was not statistically significant. Genomic characterization may improve the identification of influenza viruses that circulate postinfluenza peak.

## 1 Introduction

Influenza activity in the United States (US) in the 2022–23 season peaked earlier than any season since the 2009 influenza pandemic, with rapid increases in detections in early October 2022 [1]. Outpatient influenza‐like illness was above the national baseline for the period October 2022 through March 2023 with peak activity during the week ending November 26, 2022 [1]. This rapid, early increase coincided with many annual influenza vaccination campaigns, including those of the US Centers for Disease Control and Prevention (CDC) and the National Foundation for Infectious Diseases [2]. CDC recommends that providers offer vaccination in September or October and continue for as long as influenza viruses are circulating [3]. The season was marked by high hospitalization rates among children and adolescents [4]. Influenza A(H3N2) viruses cocirculated with influenza A(H1N1)pdm09 throughout the US; influenza B/Victoria activity was lower and occurred later in the season. In this report, we estimate influenza vaccine effectiveness (VE) against outpatient medically attended, laboratory‐confirmed influenza from December 2022 to April 2023 and describe the genetic HA clades causing disease during postpeak influenza circulation in the United States.

## 2 Methods

The US Flu VE Network has estimated seasonal influenza VE annually since the 2004–05 season [5]. In the 2022–23 season, patients were enrolled at outpatient (including emergency department) facilities associated with healthcare systems in seven states including three newly participating sites (Arizona, Missouri, and Ohio) and four that have participated since 2011–12 (Michigan, Pennsylvania, Texas, and Washington). Duke University serves as a data coordinating center. Duke University Institutional Review Board (IRB) approved the study; individual site and CDC ethical review boards relied on Duke University. Enrollment began upon IRB approval of revised US Flu VE Network protocols (Table S1).

The network enrolled participants aged 6 months or older with an acute respiratory illness (ARI) that included new or worsening cough who presented for care ≤ 7 days after illness onset. Following informed consent, study staff interviewed participants or their parent/guardian to obtain demographic and clinical information, including receipt of the current season influenza vaccine. The presence of ≥ 1 prespecified underlying medical conditions were defined based on International Classification of Diseases (ICD‐10) codes as previously described [6]. Participant vaccination status was obtained using electronic health records, state immunization registries, or plausible self‐report as previously described [6]. All participants were classified as vaccinated if they had received ≥ 1 dose of any licensed influenza vaccine product after July 1, 2022.

Participants with respiratory specimens that tested positive for influenza by RT‐PCR were classified as cases. Participants who tested positive for SARS‐CoV‐2 by RT‐PCR were excluded [7]. Influenza‐positive specimens were genetically characterized using next generation sequencing locally (five sites) or at CDC (two sites). Each sample's HA clade was based on its consensus sequence [8]. US virologic surveillance data were used for comparisons of influenza virus distributions by state during the study enrollment period [1]. HA clade distributions were compared using Chi‐square tests with *P*‐values < 0.05 considered statistically significant.

Influenza VE was estimated overall and stratified by age from logistic regression models adjusted a priori for participant age and study site as ((1–adjusted odds ratio) × 100%) comparing vaccination among cases versus test‐negative control participants. We applied Firth's method of penalization due to small sample sizes. Adjusted estimates were not calculated when the number of cases was fewer than 25. Analyses were conducted using SAS Version 9.4 (SAS Institute, Cary, North Carolina, USA) or R Version 4.3 (R Foundation, Vienna, Austria).

## 3 Results

Influenza virus detections peaked locally at each network site between weeks ending November 12 and December 17, 2022 (Table S1). Enrollment of 3095 participants took place between December 12, 2022, and April 30, 2023 (Figure S1).

Of the 2561 included participants, 91 (4%) tested positive for influenza—47 A(H1N1)pdm09, 24 A(H3N2), 6 B/Victoria, and 16 influenza A with subtype undetermined—and 2 tested positive for both influenza A(H3N2) and A(H1N1)pdm09. Among study participants, distributions of influenza cases and test‐negative controls differed by self‐reported race and ethnicity, and study site; vaccinated versus unvaccinated patients differed by patient characteristics, presence of underlying medical conditions, and self‐reported general health (Table 1).

**TABLE 1:** Characteristics of participants enrolled in the US Influenza Vaccine Effectiveness Network for the 2022–23 influenza season.^a^

Characteristic	Total no. of patients	Test result status		*P*‐value	Vaccinated	*P*‐value
		Influenza‐positive	Influenza‐negative			
		No. (row %)	No. (row %)		No. (row %)	
Overall	2561	91 (4)	2470 (96)	—	1161 (45)	—
Study site						
Arizona	110	2 (2)	108 (98)	<0.01	24 (22)	<0.01
Michigan	156	3 (2)	153 (98)		95 (61)	
Missouri	319	34 (11)	285 (89)		154 (48)	
Ohio	98	3 (3)	95 (97)		30 (31)	
Pennsylvania	535	25 (5)	510 (95)		298 (56)	
Texas	1081	24 (2)	1057 (98)		410 (38)	
Washington	262	0 (0)	262 (100)		150 (57)	
Sex^b^						
Male	928	37 (4)	891 (96)	0.38	392 (42)	0.02
Female	1630	54 (3)	1576 (97)		768 (47)	
Age group						
6 months–8 years	441	15 (3)	426 (97)	0.87	129^c^ (28)	<0.01
9–17 years	221	8 (4)	213 (96)		78 (35)	
18–49 years	1084	37 (3)	1047 (97)		423 (39)	
50–64 years	429	19 (4)	410 (96)		246 (57)	
≥ 65 years	386	12 (3)	374 (97)		285 (74)	
Race/ethnicity^d^						
White or Caucasian	1379	39 (3)	1340 (97)	0.01	701 (51)	<0.01
Black or African American	558	33 (6)	525 (94)		206 (37)	
American Indian, Alaska Native, Asian, Native Hawaiian, or other Pacific Islander	185	5 (3)	180 (97)		74 (40)	
Hispanic	349	10 (3)	339 (97)		142 (41)	
Self‐rated health general status^e^						
Fair/poor	339	13 (4)	326 (96)	0.91	179 (53)	<0.01
Good	767	29 (4)	738 (96)		412 (54)	
Very good	786	25 (3)	761 (97)		343 (44)	
Excellent	662	24 (4)	638 (96)		222 (34)	
Presence of ≥1 underlying health conditions^f^	1376	51 (4)	1325 (96)	0.65	766 (56)	<0.01
Illness onset to enrollment (days)						
0–2	955	45 (5)	910 (95)	0.05	414 (43)	<0.01
3–4	904	26 (3)	878 (97)		383 (42)	
5–7	702	20 (3)	682 (97)		364 (52)	
Receipt of COVID‐19 vaccine since September 1, 2022^g^						
Yes	958	23 (2)	935 (98)	0.01	618 (65)	<0.01
No	1525	66 (4)	1459 (96)		498 (33)	
COVID‐19 rapid test before enrollment^h^						
Yes	742	26 (4)	716 (96)	0.92	444 (60)	<0.01
No	1814	65 (4)	1749 (96)		714 (39)	
Influenza test result						
Negative	2470	—	2470	—	1126 (46)	—
Influenza A positive^i^	85	85	—		32 (38)	
A(H1N1)pdm09	47	47	—		16 (34)	
A(H3N2)	24	24	—		12 (50)	
A subtype undetermined	16	16	—		5 (31)	
Influenza B/Victoria positive	6	6	—		3 (50)	

^a^ Included patients with medically attended acute respiratory illness enrolled in the US Influenza Vaccine Effectiveness Network between December 12, 2022, and April 30, 2023.

^b^ Sex was missing for three test‐negative controls.

^c^ Among children aged 6 months–8 years who were vaccinated, 77 (60%) received the recommended number of doses for the season based on their vaccination history and were considered fully vaccinated [3]. The remaining 52 (40%) vaccinated children in this age group received only one of two recommended doses and were considered partially vaccinated.

^d^ Race/ethnicity was missing for 4 influenza‐positive cases and 86 test‐negative controls.

^e^ Participant‐rated general health status was missing for seven test‐negative controls.

^f^ High‐risk conditions included chronic cardiac diseases and circulatory diseases, chronic pulmonary diseases, diabetes mellitus, chronic renal disease, hemoglobinopathies, immunosuppressive disorders, malignancy, metabolic diseases (excluding diabetes mellitus), liver diseases, neurological/musculoskeletal conditions, cerebrovascular disease, morbid obesity, and endocrine disorders.

^g^ Participant or parent/guardian report of receipt of ≥ 1 dose of any COVID‐19 vaccine since September 1, 2022. Responses were missing for 2 cases and 76 test‐negative controls.

^h^ Participants were asked whether they had done a rapid at‐home COVID‐19 antigen testing prior to seeking care for the current illness. Responses were missing from five test‐negative controls.

^i^ Two participants tested positive for both influenza A(H1N1)pdm09 and A(H3N2). They are counted for each subtype.

Receipt of a 2022–23 influenza vaccine was documented in electronic immunization records for 1052 (91%) of 1161 vaccinated participants with a plausible self/parent/guardian report with a date and location of vaccination; 109 participants without documented vaccination reported receiving an influenza vaccine ≥ 14 days before illness onset. Over half of vaccinated children aged 6 months to 8 years (77/129, 60%) received the recommended number of vaccine doses for the 2022–23 season based on their vaccination history [3]. Vaccinated patients were significantly more likely than unvaccinated patients to also report COVID‐19 vaccination since September 2022 and were more likely to report testing for COVID‐19 at home prior to seeking care. Vaccinated influenza‐positive cases had a shorter median interval between vaccination and illness onset (83 days, IQR 48–128) than vaccinated influenza‐negative controls (129 days, IQR 93–162; *p* < 0.01).

Overall, 35 (38%) of 91 influenza‐positive participants and 1126 (46%) of test‐negative participants enrolled during the study period were vaccinated (Table 2). After adjustment for participant age and site, VE against medically attended outpatient influenza was 30% (95% confidence interval [CI]: −9% to 54%). Age group‐specific estimates of VE against any influenza were also not statistically significant. VE against influenza A(H1N1)pdm09‐associated illness was 47% (95% CI: 1 to 72); adjusted VE estimates against influenza A(H3N2) and B/Victoria during the study period were not calculated due to insufficient sample size. Results were similar when test‐negative participants from Washington, where no influenza‐positive cases were enrolled during this time, were excluded.

**TABLE 2:** Influenza vaccine effectiveness in the 2022–23 influenza season against all influenza and by influenza A subtype, December 2022–April 2023.

Influenza type/age group	Influenza‐positive		Influenza‐negative		Adjusted vaccine effectiveness^a^ % (95% CI)
	Total	Vaccinated no. (%)	Total	Vaccinated no. (%)	
Overall	91	35 (38)	2470	1126 (46)	30 (−9 to 54)
6 months–17 years	23	4 (17)	639	203 (32)	NR
≥ 18 years	68	31 (46)	1831	923 (50)	23 (−29 to 54)
Influenza A	85	32 (38)	2208	976 (44)	34 (−6 to 58)
Influenza A(H1N1)pdm09^b^	47	16 (34)	2113	946 (45)	47 (1 to 72)
Influenza A(H3N2)^c^	24	12 (50)	2005	922 (46)	NR

Abbreviations: CI, confidence interval; NR, not reported due to insufficient number of cases.

^a^ Adjusted for site and age (age group as < 18 years vs ≥ 18 years; age continuously in years for age group‐specific estimates).

^b^ Does not include participants from Washington or Ohio sites due to lack of influenza A(H1N1)pdm‐09‐positive detections during this period.

^c^ Does not include participants from Washington, Arizona, or Ohio sites due to lack of influenza A(H3N2)‐positive detections during this period.

**FIGURE 1:**
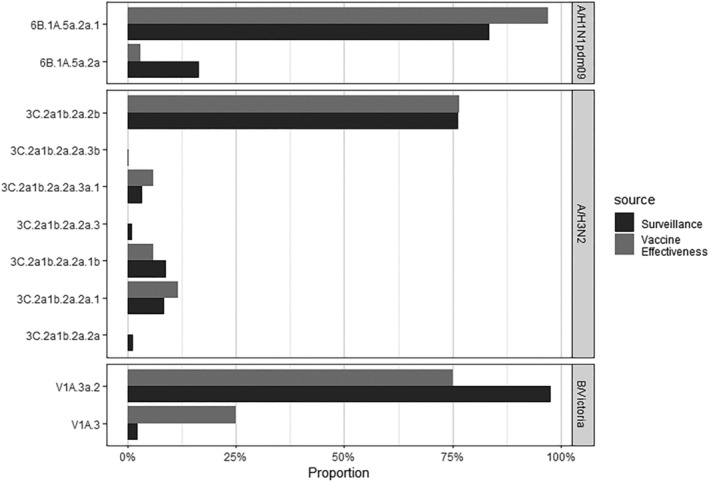
Distributions of influenza A(H1N1)pdm09, A(H3N2), and B/Victoria HA clades among US viral surveillance data from US Flu VE Network states (*N* = 2304) versus sequenced US Flu VE Network specimens (*N* = 55), December 2022–April 2023.

Among 55 (60%) influenza‐positive specimens sequenced, A(H1N1)pdm09 viruses belonged mainly to HA clade 6B.1A.5a.2a.1 (*n* = 33, 97%), with one 6B.1A.5a.2a (3%); A(H3N2) viruses belonged to HA clades 3C.2a1b.2a.2b (*n* = 13, 76%), 3C.2a1b.2a.2a.1 (*n* = 2, 12%), 3C.2a1b.2a.2a.1b (*n* = 1, 6%), and 3C.2a1b.2a.2a.3a.1 (*n* = 1, 6%); and B/Victoria viruses belonged to HA clades V1A.3a.2 (*n* = 3, 75%) and V1A.3 (*n* = 1, 25%) (Figure 1). No difference in HA clade distributions was observed by influenza vaccination status in these samples (data not shown).

Nationally, a greater proportion of A(H1N1)pdm09 viruses was observed during the period of study enrollment from December 2022 to April 2023 (31% of sequenced viruses) than during the preceding period including peak influenza activity (25% of sequenced viruses). The distributions of HA clades observed in VE samples were similar to the distributions of HA clades observed in CDC's virologic surveillance from the same time period and states (Figure 1). During the study enrollment period, 2304 influenza viruses from states with US Flu VE Network sites that detected cases were sequenced as part of US virologic surveillance. Among 630 of the sequenced A(H1N1)pdm09 viruses, 83% belonged to HA clade 6B.1A.5a.2a.1 with 17% belonging to HA clade 6B.1A.5a.2. Among 1588 sequenced A(H3N2) viruses, HA clade 3C.2a1b.2a.2b predominated (76%) with some detection of 3C.2a1b.2a.2a.1 (9%) and 3C.2a1b.2a.2a.1b (9%) viruses. Among 86 sequenced B/Victoria viruses, HA clade V1A.3a.2 predominated (98%).

## 4 Discussion

In a study conducted after the peak influenza activity, the VE point estimate against medically attended laboratory‐confirmed influenza was within the range of other estimates of 2022–23 season VE that included data collected during peak influenza activity when A(H3N2) viruses predominated in the United States [9, 10, 11]. During this postpeak study period, a majority of case patients had A(H1N1)pdm09‐associated illness. Nearly all sequenced A(H1N1)pdm09 viruses belonged to the 6B.1A.5a.2a.1 HA clade, a descendent of clade 6B.1A.5a.2 viruses that included the 2022–23 A(H1N1)pdm09 vaccine component, A/Victoria/2570/2009. The VE against A(H1N1)pdm09 observed in this study (47%, 95% CI: 1–72) is within the range (28%–56%) of the few other estimates of VE against A(H1N1)pdm09 viruses from the season [11, 12, 13]. The 2023–24 Northern Hemisphere A(H1N1)pdm09 vaccine component was updated to include an A/Victoria/4897/2022‐like virus representing the 6B.1A.5a.2a.1 HA clade [1].

This study enrolled few A(H3N2)‐positive cases, and we were unable to detect statistically significant VE. Overall US viral surveillance data and early 2022–23 season reports from other VE studies noted a predominance of A(H3N2) viruses [1, 13, 14, 15]; most published interim influenza VE estimates were against A(H3N2) or combined influenza A virus‐associated illness (A subtypes combined). VE against A(H3N2) was estimated to be ≥ 45% midseason in other VE studies [12, 13, 14, 15]. These differences highlight the ever‐changing influenza viral landscape throughout the season, the importance of postpeak surveillance, and the value of genetic characterization of influenza viruses.

This study is subject to at least three limitations. We enrolled few influenza‐positive cases because enrollment occurred after the peak of the US influenza season, which limited our statistical power and resulted in imprecise influenza A subtype‐specific estimates and precluded clade‐specific estimates. In addition, this analysis did not address the potential waning of vaccine protection after peak influenza activity at study sites. However, midseason VE estimates from January to February 2023 from other platforms reported similar VE [12, 13]. Finally, unmeasured confounding in observational test‐negative studies may bias VE estimates.

Annual influenza circulation patterns, including when influenza activity will begin increasing and when activity will peak, are difficult to predict in advance. Earlier and extended periods of enrollment in VE surveillance platforms throughout periods of local influenza circulation are important for influenza VE surveillance efforts because circulating strains and resulting VE may vary if new subvariants emerge. These findings may have implications for the influenza vaccine strain selection process.

## Author Contributions

**Jessie R. Chung:** conceptualization, investigation, writing–original draft, formal analysis. **Philip Shirk:** investigation, writing–review and editing, formal analysis, data curation, visualization. **Manjusha Gaglani:** conceptualization, investigation, writing–review and editing, data curation. **Manohar B. Mutnal:** conceptualization, investigation, writing–review and editing, data curation. **Mary Patricia Nowalk:** conceptualization; investigation, writing–review and editing, data curation. **Krissy Moehling Geffel:** conceptualization, investigation, writing–review and editing, data curation. **Stacey L. House:** conceptualization, investigation, writing–review and editing, data curation. **Tara Curley:** conceptualization, investigation, writing–review and editing, data curation. **Karen J. Wernli:** conceptualization, investigation, writing–review and editing, data curation. **Erika L. Kiniry:** conceptualization, investigation, writing–review and editing, data curation. **Emily T. Martin:** conceptualization, investigation, writing–review and editing, data curation. **Ivana A. Vaughn:** conceptualization, investigation, writing–review and editing, data curation. **Vel Murugan:** conceptualization, investigation, writing–review and editing, data curation. **Efrem S. Lim:** conceptualization, investigation, writing–review and editing, data curation. **Elie Saade:** conceptualization, investigation, writing–review and editing, data curation. **Kiran Faryar:** conceptualization, investigation, writing–review and editing, data curation. **Olivia L. Williams:** conceptualization, investigation, writing–review and editing, data curation. **Emmanuel B. Walter:** conceptualization, investigation, writing–review and editing, data curation. **Ashley M. Price:** conceptualization, investigation, writing–review and editing, data curation. **John R. Barnes:** conceptualization, investigation, writing–review and editing. **Juliana DaSilva:** conceptualization, investigation, data curation, writing–review and editing. **Rebecca Kondor:** conceptualization, investigation, writing–review and editing, data curation. **Sascha Ellington:** conceptualization, investigation, writing–review and editing, data curation, supervision. **Brendan Flannery:** conceptualization, investigation, writing–review and editing, writing–original draft, supervision, data curation.

## Conflicts of Interest

EBW has received research funding from Pfizer, Moderna, Seqirus, Najit Technologies, and Clinetic for the conduct of clinical research studies. He has also received support as an advisor to Vaxcyte, consultant to ILiAD Biotechnologies, and DSMB member for Shionogi. MPN and KMG have received research grant funding from Sanofi Pasteur not related to this work. MG has received research grant funding from CDC, Vanderbilt University, Westat, and Abt Associates not related to this work. SLH has received funding from Seegene Inc., Abbott, Healgen, Roche, CorDx, Hologic, Cepheid, Janssen, and Wondfo Biotech not related to this work. EAS has received funding from Protein Sciences Corporation and Johnson and Johnson not related to this work. KAF has received funding from the Gilead FOCUS program, the Cuyahoga County Board of Health, and the Ohio Department of Health.

5

### Peer Review

The peer review history for this article is available at https://www.webofscience.com/api/gateway/wos/peer‐review/10.1111/irv.13342.

## Supporting information


**Table S1**Enrollment periods for each contributing site.

## Data Availability

The data that support the findings of this study are available on request from the corresponding author. The data are not publicly available due to privacy or ethical restrictions.
