# Supplementary material for: Late‐Season Influenza Vaccine Effectiveness Against Medically Attended Outpatient Illness, United States, December 2022–April 2023
Source: Influenza Other Respir Viruses. 2024 Jun 23;18(6):e13342. doi: 10.1111/irv.13342 (PMC11194453; doi:10.1111/irv.13342)

**Supplemental Table 1**. Enrollment periods for each contributing site.

| **Network site** | **Enrollment start date** | **Enrollment end date** | **Peak Week of Influenza Detection^a^** |
| --- | --- | --- | --- |
| Arizona – Arizona State University, Phoenix Childrens Hospital, Valleywise Health | February 13, 2023 | April 28, 2023 | December 3, 2022 |
| Michigan – Henry Ford Health and University of Michigan | February 10, 2023 | April 28, 2023 | December 17, 2022 |
| Missouri – Washington University at St. Louis, Barnes-Jewish Hospital | December 12, 2022 | April 8, 2023 | December 3, 2022 |
| Ohio – University Hospitals Cleveland and Cleveland Veterans Health Administration | February 6, 2023 | April 24, 2023 | December 3, 2022 |
| Pennsylvania – University of Pittsburgh, Children’s Hospital of Pittsburgh | January 19, 2023 | April 30, 2023 | December 3, 2022 |
| Texas – Baylor Scott & White Health | December 19, 2022 | April 28, 2023 | November 12, 2022 |
| Washington – Kaiser Permanente Washington | February 14, 2023 | April 28, 2023 | December 3, 2022 |

^a^ Week ending date. The peak week of influenza detection was defined as the week with the greatest number of positive clinical influenza tests in the state as reported to CDC: <https://gis.cdc.gov/grasp/fluview/fluportaldashboard.html>.

**Supplemental Figure 1**. Summary of exclusions analyses, US Flu VE Network, 2022–23 season


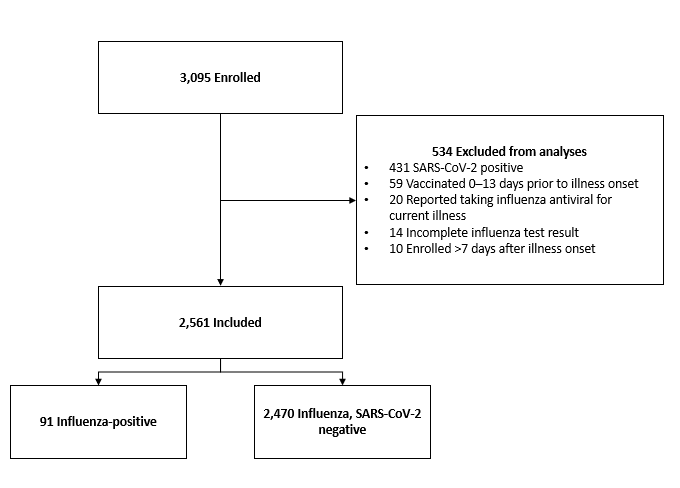

Supplement: Supplementary file 1 — Table S1Enrollment periods for each contributing site. [file IRV-18-e13342-s001.docx]
